# Room and High Temperature Tensile Responses of Tib_2_-Graphene Al 7075 Hybrid Composite Processed through Squeeze Casting

**DOI:** 10.3390/nano12183124

**Published:** 2022-09-09

**Authors:** N. Mathimurugan, V. Vaishnav, R. Praveen Kumar, P. Boobalan, S. Nandha, Venkatesh Chenrayan, Kiran Shahapurkar, Vineet Tirth, Ibrahim M. Alarifi, Moutaz Mustafa A. Eldirderi, Khaled Mohamed Khedher, Hadee Mohammed Najm

**Affiliations:** 1 Department of Mechanical Engineering, PSG College of Technology, Coimbatore 641014, India; 2 Department of Mechanical Engineering, School of Mechanical, Chemical and Materials Engineering, Adama Science and Technology University, Adama 1888, Ethiopia; 3 Mechanical Engineering Department, College of Engineering, King Khalid University, Abha 61421, Saudi Arabia or; 4 Research Center for Advanced Material Science (RCAMS), King Khalid University, Abha 61413, Saudi Arabia; 5 Department of Mechanical and Industrial Engineering, College of Engineering, Majmaah University, Al-Majmaah, Riyadh 11952, Saudi Arabia; 6 Department of Chemical Engineering, College of Engineering, King Khalid University, Abha 61421, Saudi Arabia; 7 Department of Civil Engineering, College of Engineering, King Khalid University, Abha 61421, Saudi Arabia; 8 Department of Civil Engineering, High Institute of Technological Studies, Mrezgua University Campus, Nabeul 8000, Tunisia; 9 Department of Civil Engineering, Zakir Husain Engineering College, Aligarh Muslim University, Aligarh 202002, India

**Keywords:** TiB_2_, graphene, tensile strength, squeeze casting, grain size, dislocation density

## Abstract

The development of aluminium composite with the inclusion of advanced materials is a continuous research process due to the increasing industrial demand for advanced hybrid materials. To cater for this need, this research work focuses on the development of Al 7075 alloy reinforced with TiB_2_ and graphene and on the evaluation of its strengthening mechanism. Two different modes of improving the strength of the hybrid composite have been followed; one is by the inclusion of graphene at three levels of 0.1, 0.2 and 0.3%, and another by the processing route, squeeze casting technique by compression of the molten hybrid composite slurry before casting. The microstructure and characterisation of the composite material are examined and analysed with the help of XRD, SEM, EDAX and chemical spectroscopy. A microstructure evaluation is employed to justify the homogenous dispersal and the existence of reinforced particles. A tensile test is conducted at room temperature and high temperature environments to assess the tensile strength. The research outcome affirms that a significant improvement in tensile and hardness has been noted in comparison with base alloy. The fracture-morphology results affirm the change in fracture mode from brittle to ductile when the tensile testing environment changes from room temperature to high temperature. Finally, the dispersion strengthening mechanism is validated with an empirical approach.

## 1. Introduction

There are extensive research outcomes and initiations in developing novel metal matrix composites. The recent challenges in the product development to meet the requirement of higher specific strength with minimal weight pave the opportunities for the consistent research in the development of metal matrix composite. In particular, the automobile and aircraft industries mainly focus on and encourage the development of novel metal matrix composite [1,2,3,4]. The reinforcement of hard particles in the form of carbides (TiC, SiC, B_4_C) [5,6,7,8], oxides (Al_2_O_3,_ MgO, ZrO_2_, ZrSiO_4_) [9], borides (TiB_2_, AlB_2_) [10] and nitrides (BN, AIN) [11] have reported significant improvement in the mechanical properties. However, a unique nanomaterial with a two-dimensional structure derived from graphite called graphene possesses incredible properties such as high thermal conductivity (5.30 × 10^3^ W/mK) [1,12,13], Youngs modulus (1 TPa) [12], intrinsic strength (130 GPa) and higher surface area (2600 m^2^/g) [13]. Few researchers [14,15,16] reported the improvement of mechanical properties in multiple folds with the inclusion of graphene. Aluminium is the most promising matrix material due to its abundant availability, low cost, lesser weight and the easiness of its processing in any route. There are remarkable research outcomes [17,18] stating the benefit of including more than one reinforcement to form a hybrid composite. Apart from reinforcing materials either in single or hybrid, the processing route of aluminium composite is also a key factor in determining the enhancement of properties. Usually, AMC are manufactured in two broad ways, namely solid-state (diffusion bonding, friction stir processing, powder metallurgy) and liquid-state (stir casting, squeeze casting, centrifugal casting) processing [19]. Generally, most of the methods produce the composite with inhomogeneous reinforcement, voids and coarse grain structure. However, the squeeze casting with melt stirring is one of the versatile processing routes that impart the composites with zero porosity and fine grain structure [20]. Bi Jiang et al. [21] developed the Al 7075 matrix reinforced with TiB_2_ composite through laser melting deposition, and they subsequently concluded that the grain size was reduced to a considerable extent and the mechanical properties also improved to a greater extent. Han Wang et al. [22] investigated the compressive response and microstructure improvement of TiB_2_ reinforced Al 7075 matrix composite, and the authors concluded that the grain size of the composite is smaller than the Al 7075 base alloy. The inhibit property of TiB_2_ to promote nucleation is the reason for the grain growth. B.P. Sahoo et al. [10] validated the strengthening mechanism of submicron TiB_2_ particles infused Al 7075 matrix through experimental and theoretical approach. The research outcome states that nearly 58% of grain size reduction is achieved with the higher content of TiB_2_ and in turn the noticeable improvement of mechanical properties. Vineet Chak et al. [23] synthesized the graphene aluminium composite and studied the mechanical and tribological properties. The authors reported that the tensile strength and hardness of the composites increased by 50% more than the base alloy due to the grain size improvement. Subsequently, the coefficient of friction is reduced to the minimal level owing to the self-lubricating property of graphene. Rangaraj et al. [24] studied the effect of graphene inclusion over the Al 7075 and declared that the hardness, tensile strength and tribological properties are increased with the increased level of graphene. Prashanth Kumar et al. [25] concluded the homogenous inclusion of silicon carbide and graphene in aluminium alloy through ball milling. Bozic et al. [26] carried out a compressive fracture behaviour of CW67 aluminium alloy reinforced with SiC under uniaxial compressive loading in the temperature range of 400 °C at a constant strain rate. The authors declared that there is a significant improvement in yield strength at all temperatures in comparison with the monolithic alloy. Hassan et al. [27] developed magnesium composite reinforced with Al_2_O_3_ through liquid melt deposition followed by hot extrusion and conducted characterization, high temperature tensile and fracture study. They reported that the maintained yield strength up to 150 °C and the fracture analysis revealed the transition failure from brittle to ductile.

Numerous research reports that address the improvement of properties of aluminium alloy with the reinforcement of various hard particles are available. However, very scarce research was conducted with the inclusion of graphene on Al 7075 and through the squeeze casting route. In particular, none of the work has been reported with inclusion of TiB_2_ and graphene. The objective of the present work is to advocate for a dual way of enhancing the mechanical and tribological properties of Al 7075 composite, one way being through the encapsulation of graphene with hard boride particles and another is by achieving porous-free composites through the processing route. Three different levels of graphene content are being followed to compare the effect of graphene inclusion. Additionally, very specific attention has been paid to expose the tensile response of the composite materials at room and high temperature environments. Subsequently, the fracture analysis is also being discussed with the help of micrographs.

## 2. Materials and Methods

### 2.1. Materials

Aluminium 7075 alloy is used as a matrix material and its mechanical properties are depicted in Table 1. The hard boride particle TiB_2_ purchased from Metal mart Coimbatore and the graphene flakes purchased from Shilpa enterprises are used as reinforcements. The mechanical properties of TiB_2_ and graphene are tabulated in Table 1.

### 2.2. Processing of Hybrid Composite

**Figure 1:**
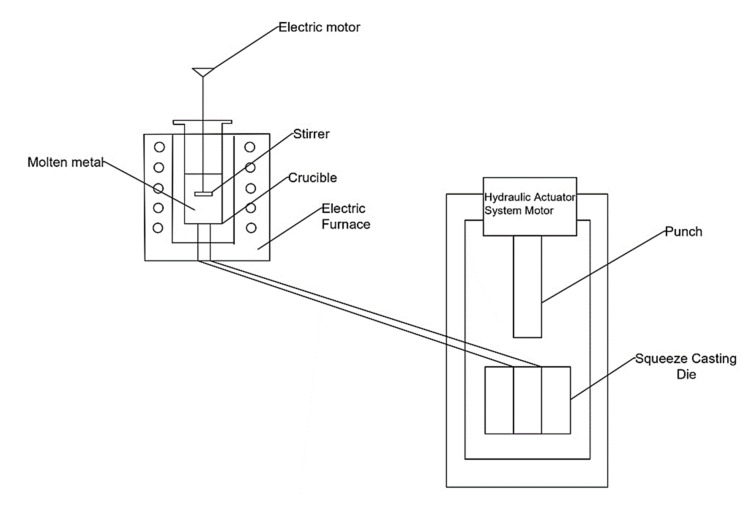
Schematic arrangement of squeeze casting setup.

Initially, the TiB_2_ particles and graphene powders with premeasured quantity were preheated to 200 °C to eliminate chemical impurities and moisture. Then, the aluminium alloy was melted to 800 °C in an electric furnace in an open atmosphere. The present work followed constant weight percentage of TiB_2_ as 10% [21,28,29] and three variational weight proportions of graphene as 0.1%, 0.2% and 0.3% [20] to fabricate the three different specimens. A table showing the included amount of TiB_2_ and graphene in each sample is presented in Table 2. The premeasured and preheated reinforcement particles were added to the liquid melt. The liquid melt was stirred at 525 rpm with the stainless-steel mechanical stirrer coated with graphite. The blade angle was set to 30° to attain the maximum homogeneity, and the stirring time was maintained at 10 min [25]. An inert atmosphere was developed with a small addition of Mg with the melt to enhance the wettability. To enhance grain refinement during the solidification of castings, a small quantity of zirconium was added to the melt as a grain refiner [30]. The die accessories of the squeeze casting arrangement were preheated to 250 °C. After stirring, the molten slurry was transferred to preheated die and was squeezed at a pressure of 100 MPa with the help of a hydraulic punch and the compaction was held on standby for 45 s to induce the non-porous structure. The schematic arrangement of the squeeze casting set is shown in Figure 1. After the solidification, the specimens were removed from the cylindrical mould with the measurement of 50 mm in diameter and 220 mm in length. The stir cum squeeze casting parameters are furnished in Table 3.

### 2.3. Characterization

The characterization of the developed hybrid composite was carried out in four different ways. Scanning electron microscopy (SEM), chemical spectroscopy (ChemS), energy dispersive spectroscopy (EDS) and X-ray diffraction (XRD) techniques were employed to study the phase and microstructure. The specimen was cut into the size of 10 mm × 10 mm × 10 mm and subsequently rinsed in acetone followed by polishing through silicon carbide emery sheet. An SEM study was conducted using De-Wintor inverted trinocular metallurgical microscope (Delhi, India). The chemical composition and elemental analysis were executed with the help of X-ray Diffraction and chemical spectrometer from Malvern Panalytical (Cambridge, UK). The experimental density of the composite was determined with the help of a densimeter (Mettler Toledo, Mumbai, India) working under the Archimedes’s principle of displacement. The theoretical density was evaluated with the following relation of the rule of mixture Equation (1), and the percentage of porosity was calculated using the below equation Equation (2).

(1) $$\rho_{c\;}=\rho_{m}W_{m}+\rho_{r}W_{r}$$

(2) $$P=\left[1-\frac{\;\rho_{c\;}}{\;\rho_{c}\left(1-W_{r}\right)+\rho_{r}W_{r}}\right]100$$

where *ρ_m_, ρ_r_* and *ρ_c_* represent the density of matrix, reinforcement and composite, respectively. *V_m_, V_r_* indicate the weight percentage of matrix and reinforcement. Furthermore, the particle size analysis is carried out using CILAS 1064 Liquid setup.

### 2.4. Dislocation Density

The results of XRD were employed to determine the crystallite size and lattice strain. The Sherrer equation, as shown below, was utilized to calculate the crystallite size [31].

(3) $$d=\frac{k\lambda }{\beta cos\theta }$$

where, *d* is the size of the crystallite, *k* is the shape factor, *λ* is the wavelength, *θ* is Bragg angle, and *β* is the diffraction peak at half-width maximum intensity.

The lattice strain was manipulated by using the following equation, [32]

(4) $$\beta \;cos\theta =\frac{0.9\lambda }{d}+2A\;\varepsilon \;sin\theta$$

where d is the size of the crystallite, *λ* is the wavelength, *A* is constant, *θ* is Bragg angle, *β* is the diffraction peak at half-width maximum intensity, and ε is lattice strain.

Now the dislocation density can be determined for every specimen by using the following relation [33],

(5) $$\rho =\frac{\sqrt[{2}]{3\varepsilon }}{v_{b}\;d}$$

In this equation, *ρ* represents dislocation density, *ε*, *v_b_* and *d* indicate lattice strain, Burgers vector and crystallite size, respectively.

### 2.5. Tensile Test

The tensile test was conducted in two different modes: room temperature and elevated temperature tensile testing. The size of the test specimen was trimmed as per ASTM 638-03 (165 mm length × 19 mm width × 3 mm thickness). The tensile test was carried out at Associated Scientific Engineering works with a range of maximum 5-ton load. Specimens were heated up to 250 °C through an electric furnace to conduct the elevated temperature test.

### 2.6. Hardness and Flexural Test

The hardness test was conducted by following ASTM E-10 standard (25 mm × 25 mm × 10 mm) with the help of a micro-Vickers hardness tester (Wilson Wolpert, Bremerhaven, Germany), with testing load range of 10 g to 1 kg. As per ASTM D-790 standard, flexural test was carried out in the three-point bending mode. The maximum bending stress in N/mm^2^ was evaluated with the following relation,

(6) $$\sigma =\frac{My}{I}$$

where *M* is the bending moment in N mm, *Y* is the distance between the neutral axis to the extreme fibre measured in mm, and I is the moment of inertia of the section calculated in mm^4^. *M* is the bending moment, *Y* is the distance between the neutral axis to the extreme fibre, and *I* is the moment of inertia of the section.

## 3. Results and Discussion

### 3.1. Density Evaluation

**Figure 2:**
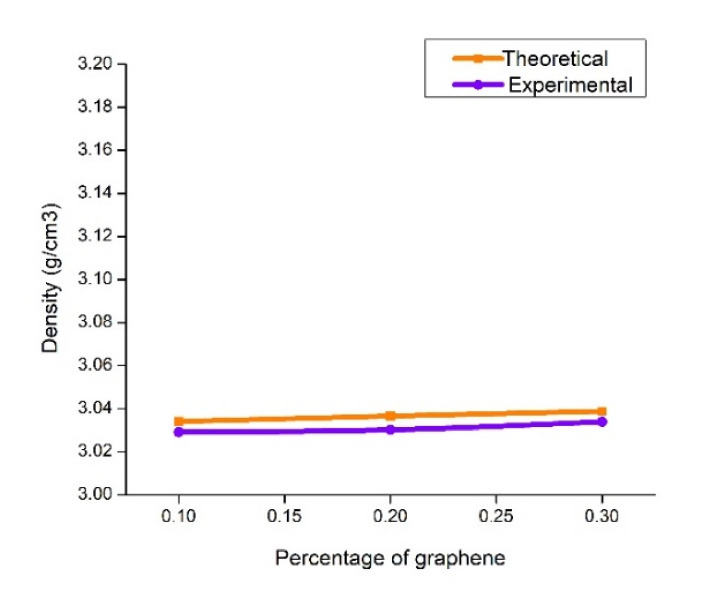
Density plot.

The deviations between the experimental and theoretical density of the composite with the TiB_2_ and graphene reinforcement is presented in Figure 2. The incremental weight percentage of graphene makes a minor increment in the density of the composite due to the density of graphene. Table 4 shows the calculated value of void percentage for each category of graphene inclusion. A good agreement between the theoretical and experimental density is observed with the close coincidence. The theoretical and experimental density difference is considered a void percentage. The table value affirms the very minimal void percentage (maximum of 0.16%) revealing the developed composite as porous-free. The improved void-free structure is attributed to the compaction effect of the squeeze casting process.

### 3.2. Characterisation of Hybrid Composite

**Figure 3:**
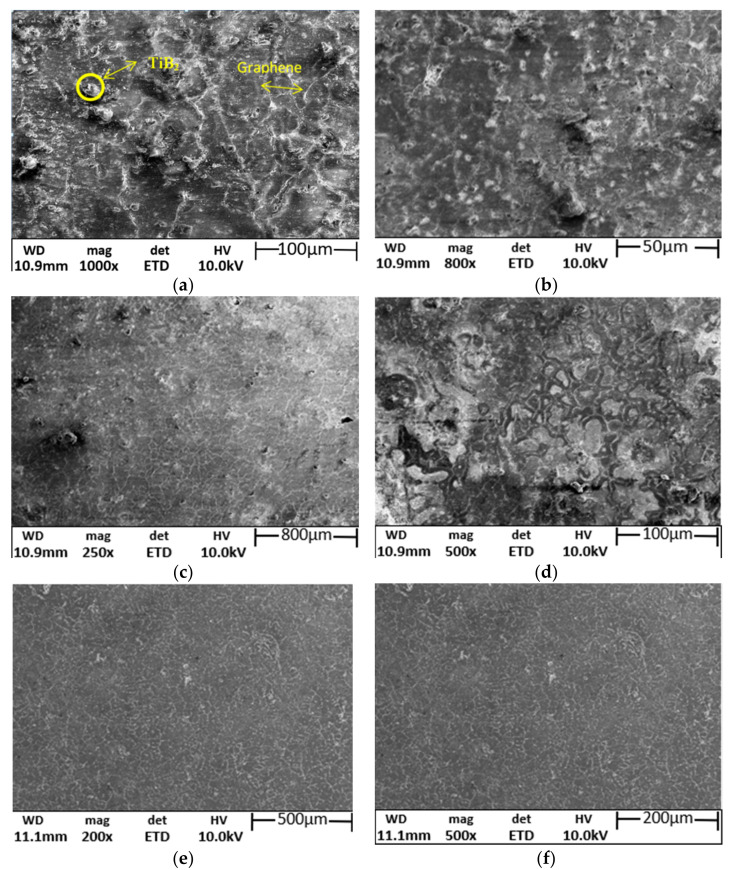
SEM images of AM hybrid composite (**a**) Reinforced particles, (**b**) Distribution of graphene particles, (**c**) Distribution of TiB2 particles, (**d**) Improved thickness of grain boundary, (**e**) Finer grain development, (**f**) Magnified version of finer grains.

Characterisation and phase identification were conducted with four different methods, namely SEM, EDS, XRD and ChemS. From SEM micrographs, it has been inferred that the homogeneity in the distribution of the two reinforcements exists. The absence of voids and porous microstructure is observed through the micrographs. The compression pressure to the extent of 100 MPa helped to relieve the voids and porosity. The EDS and chemical spectroscopy results confirm the existence of TiB_2_ particles and graphene nanoflakes. The inclusion of TiB_2_ particles is an additional factor for the enhancement of the microstructure by controlling the precipitation mechanism (Figure 3a). Excellent grain refinement is achieved and realized in Figure 3b with many heterogeneous nucleations developed by TiB_2_ particles. Figure 3d acknowledges the improved thickness of the grain boundaries developed through the rapid movement of dislocation [10]. The significant eutectic phase transformation is attained with the help of the heterogeneous nucleation characteristics of TiB_2_. Figure 3e annunciates the existence of fine grain as an outcome of the improved processing technique.

**Figure 4:**
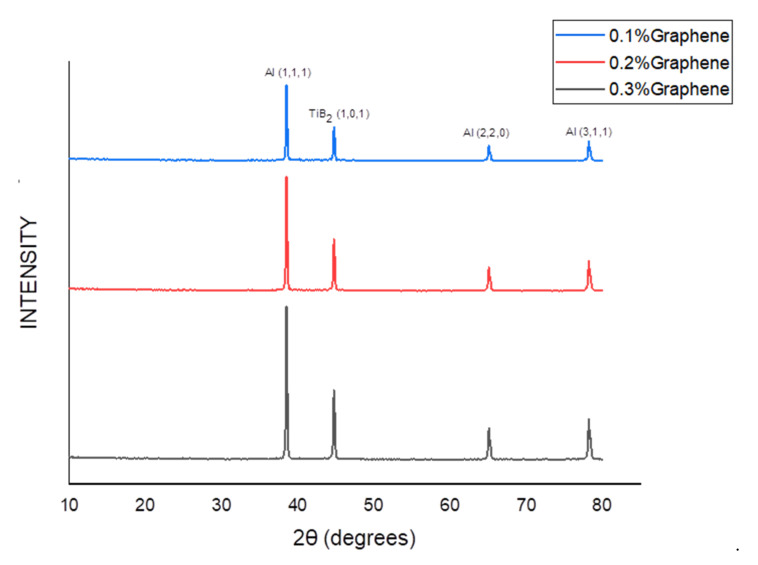
XRD results for various proportions of graphene.

**Figure 5:**
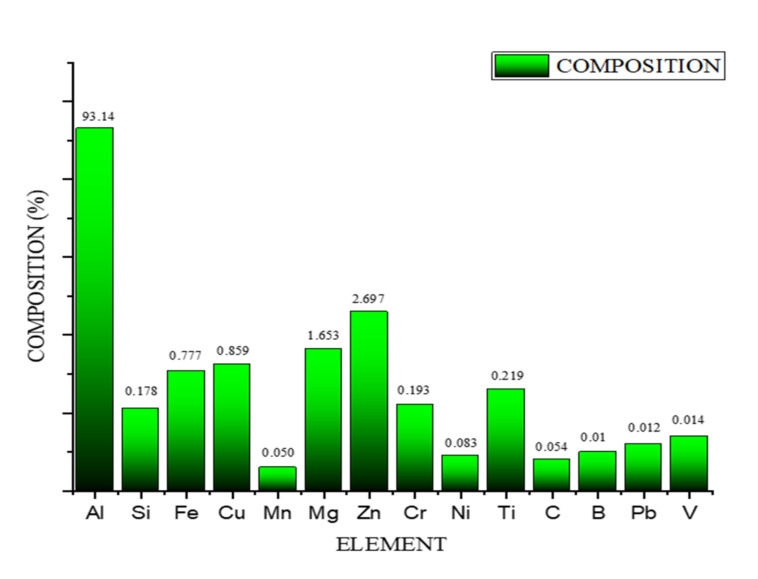
Spectroscopy results.

**Figure 6:**
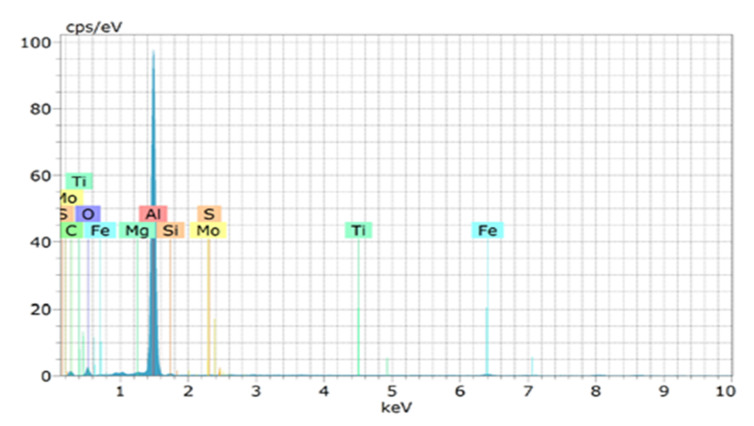
Energy dispersive X-ray spectroscopy of Al−10 % TiB_2_−0.3 % graphene.

Figure 4 depicts the XRD results, which affirm the existence of reinforced particles in the hybrid composite. The miller indices planes (h.k.l) were located to match the XRD data with the ICCD’s data (international standard diffraction data) to map the miller indices (h.k.l) planes. It has been observed that the multiple peaks are evenly distributed to form a single peak, which reflects the homogeneity of the reinforcement throughout the structure. The peaks of Al and TiB_2_ are in the position to be noticed, whereas the graphene peaks are non-noticeable due to its meagre level of inclusion. The same observation was reported by the researcher [20]. However, the existence of graphene is detected in EDS and chemical spectroscopy, as shown in Figure 5 and Figure 6, respectively.

**Figure 7:**
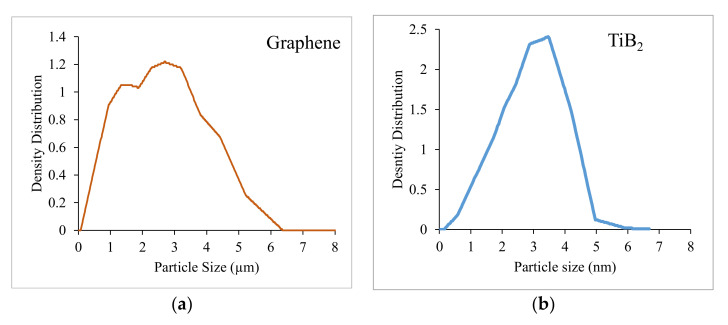
Particle size analysis of (**a**) Graphene and (**b**) TiB_2_.

Figure 7a shows the particle size analysis of the graphene particles. Weighted mean particle size is observed to be 2.7 nm. The peak for graphene particles is wider and depicts an average value of 2.85 nm. Figure 7b depicts particle size analysis of TiB_2_ particles where the weighted mean particle size is observed to be 3.9 µm. The peak for TiB_2_ particles is narrow and depicts an average value of 3.75 µm.

### 3.3. Room Temperature Tensile Strength

**Figure 8:**
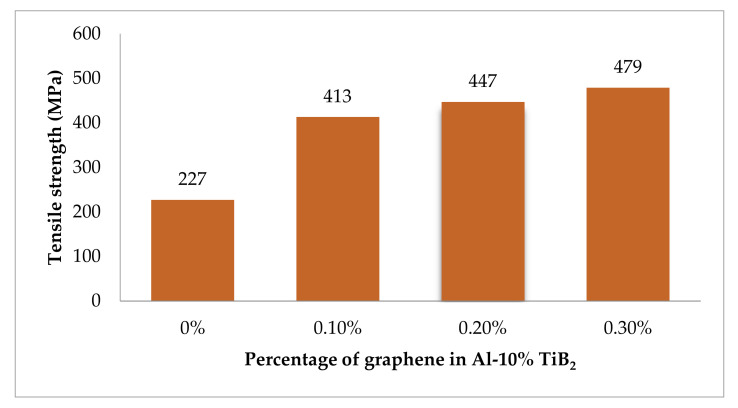
Tensile strength at room temperature.

Figure 8 shows the experimental observation of tensile strength at room temperature for the developed hybrid composite. The results infer that the rise in the percentage of graphene boosts the tensile strength by a considerable fold. The remarkable improvement in the strength of the hybrid composite is due to the distinguished strengthening mechanism developed by two different routes. One is by the inclusion of hard particles such as TiB_2_ and graphene flakes, and the other is by the way of the porous-free squeeze casting processing technique. The phenomenal increment in strength is attributed to the fine-grain structure of the hybrid composite. The noticeable enhancement in grain structure is achieved by the two modes. First, with the addition of TiB_2_ particles, the composite attains a more refined grain structure with the help of the heterogeneous nucleation capacity of TiB_2_ to form a dendrite structure [34]. Second, the processing technique involves good compaction to the extent of 100 MPa, which paves the way for finer grain growth. The existence of fine-grain structure for the composite is being validated with the SEM micrographs.

**Figure 9:**
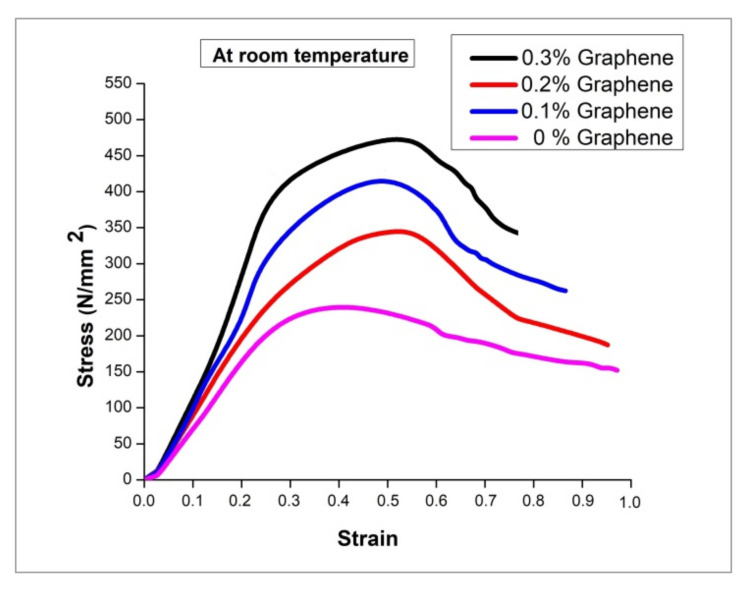
Stress-strain plot at room temperature.

Figure 9 shows the stress-strain plot for the hybrid composite with varying graphene content. The graph implies that the higher content of graphene records superior strength than the base alloy. An increase of nearly 112% in tensile strength is achieved compared to the base alloy due to the combined effect of the inclusion of particles and the processing route. Now, the strengthening mechanism can be discussed in a scientific and empirical way. The movement of dislocation during plastic deformation is viewed to be disturbed by the existence of hard particles. In particular, the graphene with a higher aspect ratio covering a large surface area to negligible thickness acts as a barrier for movement of dislocation [24], making the specimen work in a hardening state. The piling up of multiple dislocations in a single line increases the dislocation density [35]. It is well reported in various research outcomes that the Orowan mechanism plays a pivotal role in developing strength [3]. The movement of dislocation is bowing the particle and, in turn, looping the particle to form a denser dislocation [36].

### 3.4. Evaluation of Dislocation Density

The empirical prediction reveals that the increased content of graphene increases the dislocation density by resisting the impending dislocation movement. The dislocation density calculated for different proportions of graphene with an empirical approach is given in Table 5.

### 3.5. High Temperature Tensile Strength

**Figure 10:**
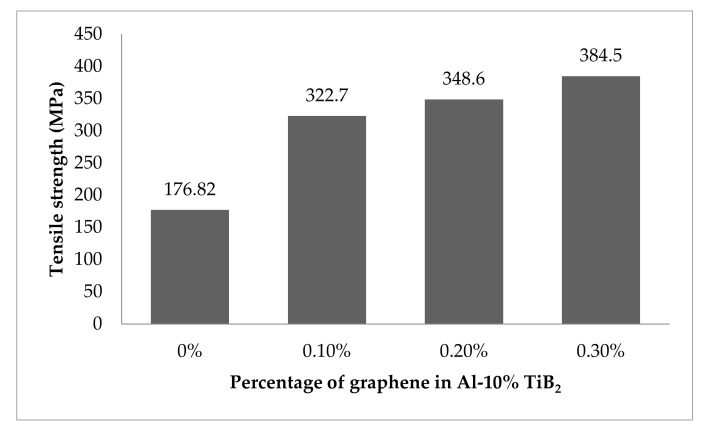
Tensile strength at high temperature.

**Figure 11:**
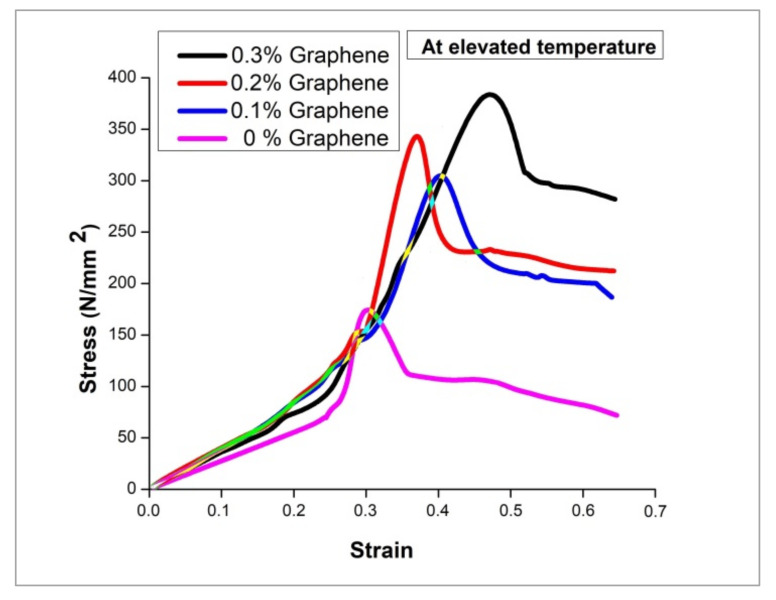
Engineering stress-strain plot at elevated temperature.

The composite specimens were fed into tensile test under an elevated temperature of 250 °C to assess the deformation behaviour in a high-temperature environment. Figure 10 depicts the results of tensile strength of specimens with varying percentages of inclusion, including base alloy in a high-temperature environment. In a high-temperature environment, also, there is no change in the trend of increasing strength concerning the graphene increment. From the results of room temperature testing, the strengthening mechanism is sufficiently discussed in terms of improved grain structure due to processing benefit, the self-nucleating capacity of TiB_2_ particles and piling up of dislocation movement into dense dislocation owing to the barrier of graphene particles. The heating of the specimens at high temperatures might cause an increment in the thickness of the oxide layer over the surface of Al particles [37]. The broken particles of oxide layers during loading might help the specimen to improve its strength. However, the stress-strain plot shown in Figure 11 at high temperature explores the change in deformation behaviour of all the specimens at thermal loading.

It is evident from the results that the percentage of elongation of all the specimens including the higher content of graphene also records a higher percentage of elongation than the specimens at room temperature. This reflects the thermal softening effect of the hybrid composite due to the high temperature. Moreover, the quick peak in strength followed by the consistent plastic deformation is attributed to the chance of diffusing dislocation density at one point in high temperature and allowing the dislocation to move freely [35]. This phenomenon makes the material more plastic, thereby improving the elongation capacity. Apart from the dislocation density diffusion, the difference in thermal expansion coefficient between reinforcement and matrix materials is also credited as that with which the material becomes more plastic. The thermal softening of the composite is attained due to a reduced level of residual stress in the matrix due to the difference in thermal expansion coefficient between reinforcement and matrix material [38].

### 3.6. Fracture Morphology

**Figure 12:**
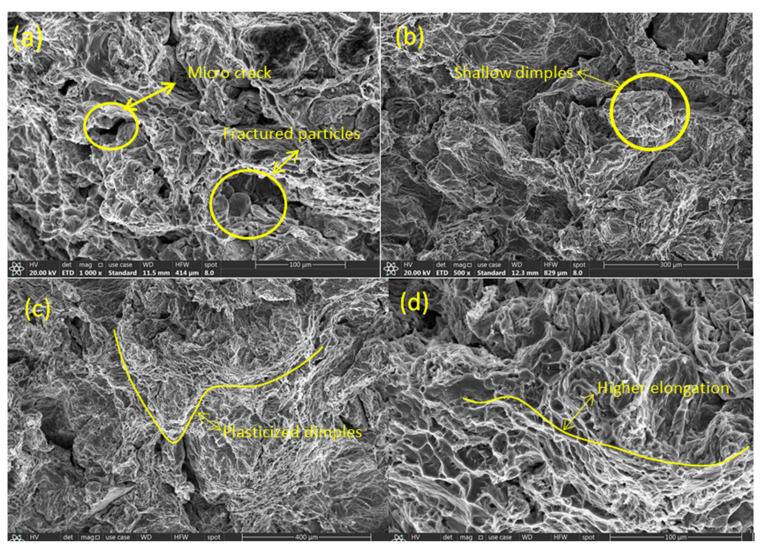
Fracture morphology (**a**,**b**) at Room temperature failures (**c**,**d**) at High temperature failures.

The investigation of fracture surface at both room temperature and high-temperature composite specimens reveals the change of failure mode with respect to the physical and thermal loading. At room temperature, the mode of failure is in a brittle manner. The plane of failure is at an angle of 90° to the axis of loading with negligible neck formation. This sudden failure is attributed to the stress concentration at the grain boundaries. When the stress value increases, the dislocations are piled up to form dislocation density due to the graphene particle inclusion. Furthermore, the stress value increases, and the stress concentration develops over the grains due to the impediment of dislocations, which initiate a micro void formation. The coalescence of micro void forms the crack, as shown in Figure 12a, with the further addition of load which makes the specimen fail. The SEM micrographs for the room temperature specimen acknowledge the existence of voids.

The dimples in more numbers with shallow depths, as shown in micrograph Figure 12b, assure the brittle nature of the failure. However, when the temperature increases, the mode of failure changes from brittle to ductile due to the thermal softening of the material. The plane of failure changes from an angle of 90° to 45° to the axis of tensile loading. The noticeable neck formation at high temperature is attributed to the increased ductile nature of the specimen. The stress concentration developed over the grains due to dislocation density is diffused at high temperature due to the diffusing behaviour of dislocation density at high temperature [39]. This phenomenon makes the composite specimen to fracture in ductile nature by registering higher elongation, as shown in Figure 12d. The coalescence of micro void and crack formation is not so rapid, thereby causing the ductile fracture. The existence of dimples in a lesser number with deep lengths observed as plasticized dimples are depicted in Figure 12c. The SEM micrographs acknowledge the change in failure mode from brittle to ductile due to the high temperature.

### 3.7. Hardness

**Figure 13:**
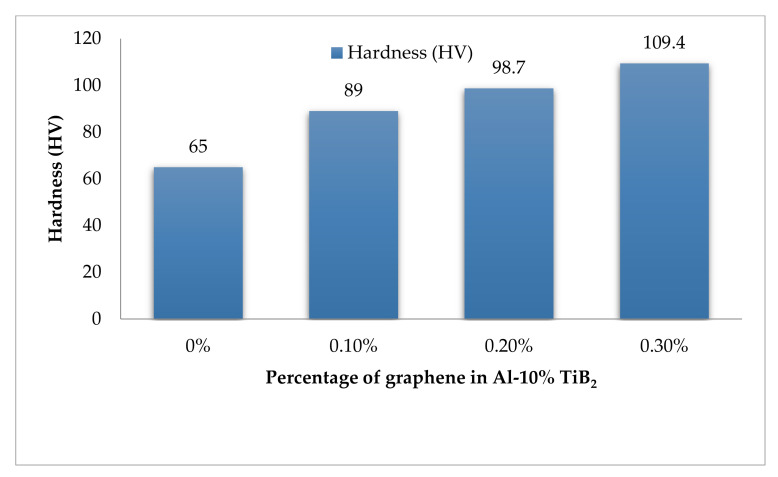
Hardness results.

Figure 13 depicts the hardness test results for three different proportions of a hybrid composite, including a base alloy. The results annunciate that the increment in graphene content increases the hardness value. The resistance to indentation due to the inclusion of hard particles such as TiB_2_ and graphene justifies the hardness value. The increment in hardness for every fold of increasing graphene content is attributed to the higher number of graphene flakes that resist the indentation due to its high aspect ratio and unique strength. The squeeze casting processing route accelerates the formation of fine-grain structure due to the consistent compaction during solidification. The hardness value is inversely proportional to the grain size advocated by Hall-Petch [40]; hence, the lesser the grain size, the higher the hardness. The SEM micrographs and theoretical modelling results confirm the existence of a fine-grain structure. The experimental evaluation of hardness corroborates the abrupt increment of the hardness of 0.3% graphene to the extent of 67.8%, in comparison to the base alloy 7075.

### 3.8. Flexural Strength

**Figure 14:**
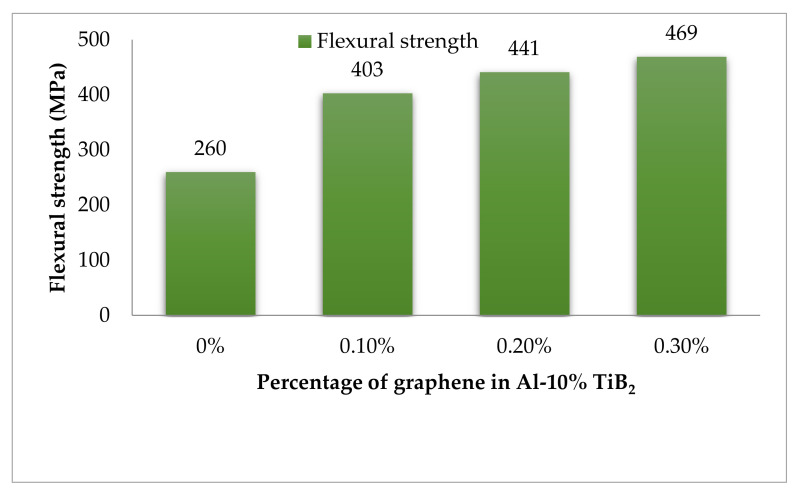
Flexural strength results.

The effect of the inclusion of TiB_2_ and graphene on the improvement in flexural strength is represented in Figure 14. From the flexural strength results, it is vivid in nature that the rise in graphene content improves the bending strength. The increased content of graphene induces the lesser bending stress owing to the strengthening mechanism discussed earlier. The three-point bending test interprets the significant jump of flexural strength for 0.3% specimen. Nearly 80% of the higher flexural strength is attained by 0.3% specimen in comparison to the base alloy.

## 4. Conclusions

1. The investigation of the tensile response of squeeze casted TiB_2_ and graphene reinforced Al 7075 hybrid composite in the room and at high-temperate environments arrives at the following conclusions.
2. The theoretical and experimental evaluation of the density of the developed composite affirms the negligence void content of 0.2%. The excellent agreement between the theoretical and experimental results of density acknowledges the porous-free nature of the hybrid composite.
3. The characterisation results earned through XRD, SEM and chemical spectroscopy confess the existence of constituent material in the homogenously dispersed state. Moreover, the improved grain structure has also been detected.
4. The room temperature tensile test conducted for the specimens with varying graphene content, including base alloy, reveals the increment in tensile strength for every fold of graphene increment. The higher graphene content of 0.3% records a drastic improvement of 112%, in comparison with the base alloy.
5. Due to the high temperature, the thermal softening effect makes the hybrid material more plastic with a noticeable increment in the percentage of elongation. The reduction in tensile strength and increment in elongation percentage are 18.5% and 28%, respectively.
6. The fracture morphology confirms the change in fracture mode from brittle to ductile, while the tensile examination changes from room temperature to high temperature. The plasticized dimples in SEM micrographs with noticeable length confirms the increment in elongation percentage, and hence the ductile failure is understood.
7. The hardness results proclaim the evident increase in hardness of higher graphene content to the extent of 67.8%, when compared to the base alloy.
8. An improvement of nearly 80% in the flexural strength of the hybrid composite has been reported, in comparison with the Al 7075 alloy, from the flexural strength results.

## Figures and Tables

**Table 1:** Mechanical properties of matrix and reinforcing materials.

Material	Density (gm/cm^3^)	Melting Point (°C)	Yield Strength (MPa)	Tensile Strength
Aluminium 7075	2.81	477	103	228 MPa
Graphene	2.267	3652	270	130 GPa
TiB_2_	5.06	3230	400	373 MPa

**Table 2:** Material nomenclature.

S.No.	Labels	Material Composition
1	0%	Al 7075 Alloy
2	0.1%	Al 7075, 10 wt.% TiB2, 0.1% Graphene
3	0.2%	Al 7075, 10 wt.% TiB2, 0.2% Graphene
4	0.3%	Al 7075, 10 wt.% TiB2, 0.3% Graphene

**Table 3:** Stir cum squeeze casting parameters.

Parameter	Value
Stirring temperature	800 °C
Stirring time	10 min
Stirrer speed	525 rpm
Preheating temperature of reinforcement	200 °C
Squeezing pressure	100 MPa
Die temperature	250 °C
Holding time in pressure	45 s

**Table 4:** Void percentage of the specimens.

% of Graphene	Theoretical Density (g/cm^3^)	Experimental Density (g/cm^3^)	Void %
0.1	3.034	3.0291	0.16
0.2	3.0365	3.0302	0.2
0.3	3.0388	3.0339	0.16

**Table 5:** Dislocation density.

Percentage of Graphene	Dislocation Density
0.1	7.6 × 1012 m^−2^
0.2	9.07 × 1012 m^−2^
0.3	3.4 × 1013 m^−2^

## Data Availability

The data used to support the findings of this study are included within the article.
